# Constructal Law of Vascular Trees for Facilitation of Flow

**DOI:** 10.1371/journal.pone.0116260

**Published:** 2014-12-31

**Authors:** Mohammad S. Razavi, Ebrahim Shirani, Mohammad Reza Salimpour, Ghassan S. Kassab

**Affiliations:** 1 Department of Mechanical Engineering, Isfahan University of Technology, Isfahan, Iran; 2 Department of Engineering, Foolad Institute of Technology, Fooladshahr, Isfahan, Iran; 3 Department of Biomedical Engineering, Indiana University–Purdue University Indianapolis (IUPUI), Indianapolis, Indiana, United States of America; 4 Department of Surgery, Indiana University–Purdue University Indianapolis (IUPUI), Indianapolis, Indiana, United States of America; 5 Department of Cellular and Integrative Physiology, Indiana University–Purdue University Indianapolis (IUPUI), Indianapolis, Indiana, United States of America; University of California San Diego, United States of America

## Abstract

Diverse tree structures such as blood vessels, branches of a tree and river basins exist in nature. The constructal law states that the evolution of flow structures in nature has a tendency to facilitate flow. This study suggests a theoretical basis for evaluation of flow facilitation within vascular structure from the perspective of evolution. A novel evolution parameter (*Ev*) is proposed to quantify the flow capacity of vascular structures. *Ev* is defined as the ratio of the flow conductance of an evolving structure (configuration with imperfection) to the flow conductance of structure with least imperfection. Attaining higher *Ev* enables the structure to expedite flow circulation with less energy dissipation. For both Newtonian and non-Newtonian fluids, the evolution parameter was developed as a function of geometrical shape factors in laminar and turbulent fully developed flows. It was found that the non-Newtonian or Newtonian behavior of fluid as well as flow behavior such as laminar or turbulent behavior affects the evolution parameter. Using measured vascular morphometric data of various organs and species, the evolution parameter was calculated. The evolution parameter of the tree structures in biological systems was found to be in the range of 0.95 to 1. The conclusion is that various organs in various species have high capacity to facilitate flow within their respective vascular structures.

## Introduction

Constructal law is a theory that stipulates the generation of design evolve structures that increase flow [1]–[3]. In 1996, Bejan stated the constructal theory as “for a finite-size flow system to persist in time (to live) its configuration must evolve in such a way that provides greater and greater access to the currents that flow through it” [4]. According to the constructal law, a living system is a non-equilibrium system in thermodynamics with a structure that morphs towards configurations that provide easier flow through the system. The constructal law hypothesizes the evolution of design [5], and a mathematical formulation of constructal law based on thermodynamics is presented in Bejan and Lorente [6]. According to the constructal law, the configuration of a flow system evolves to acquire more global performance (minimization of imperfection) over time.

Tree structures play a vital role in the transport of substance in nature. A diversity of designs with tree structures exists in nature such as blood vessels, river basins, bronchial and botanical trees. The well-known Murray's law states that the flow through a branch is proportional to the diameter cube of the branch. Murray's law represents minimization of energy dissipation, which consists of viscous dissipation and metabolic cost. This law implied (through conservation of mass) that the cube of vessels diameter at each generation is preserved, i.e., 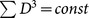. Although the HK (Huo-Kassab) model has shown the exponent is equal to 7/3 (rather than 3) in coronary branching [7], the power-law form of Murray's relation still holds in living vascular structures. Murray's law has been validated in some vascular networks in zoology [8] and in plants [9]. For instance, small arteries of the rat cardiovascular system, swine heart arterioles, and symmetrical branching pattern of leaf veins support Murray's law [10]–[13]. A comparison of the various laws that govern coronary branching including Murray, Finet and HK models are summarized in Ref. [14].

A number of studies have been conducted to deduce the design of tree structures [15]–[25]. These studies, however, have not focused on the evolutionary aspect of the constructal design. The objective of this study is to provide an analytical basis for evaluation of flow capacity within vascular structures. A novel evolution parameter (*Ev*) is proposed to evaluate the structure's capability to facilitate flow from an evolutionary perspective. The evolution parameter is obtained for tree geometries using fully developed laminar flows (Newtonian and non-Newtonian fluids) and fully rough turbulent flows. Lastly, the evolution parameter is calculated based on vascular morphometric data of various organs and species.

## Methods

### 2.1 Properties of Flow Systems

The constructal law postulates that uniform design rules of flow systems are due to universal propensity for flow facilitation in nature. According to constructal law, the configuration of flow systems evolves to provide greater access to global flow [26]. A flow system is a dynamic system with the following properties which distinguish it from a static system: 1) global external size (area occupied by a tree construct), 2) global internal size (volume of a tree construct), 3) the global performance (global flow resistance of a tree construct), 4) configuration (construction of conduits distribution on the available area or volume), and 5) freedom to morph the configuration for providing easier access to the global flow [2]. The flow system properties form dimensionless parameters that describe features of vascular design as shown below.

### 2.2 Svelteness

A flow system has a property called svelteness (*Sv*) which is the ratio between its external global and internal length scale [26]. For a symmetrical tree structure, the internal and external sizes are the volume 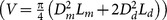 and area 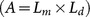 occupied by the tree structure where 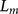 and 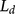, 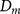 and 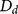, are lengths and diameters of mother and daughter branches, respectively. Consequently, the svelteness of a symmetric tree structure is expressed as [26]:
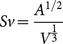
(1)The non-dimensional svelteness parameter characterizes the bulk of vessels in the design of vascular structures. Small *Sv* denotes thin vessels in the vascular system while large *Sv* denotes the converse [5]. When the body size of animal grows, the svelteness increases accordingly. Moreover, the *Sv* connects the size of a flow system to its design. It has been shown that the *Sv* has a power law relationship with the mass of system (*M*) for laminar flows 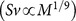 and turbulent flows 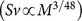 [5], [16], [27].

### 2.3 Evolution parameter

A novel dimensionless parameter is proposed for evaluating the evolution of the geometry of a vascular system. The flow through a living system along with its shape and size, are subjected to continuous change in response to physical and environmental stimuli. In view of constructal design, the configuration of a flow system must evolve to provide easier access to global flow. In other words, the time direction of evolution of a flow system, which is subject to different constraints, is in the general direction of attaining greater flow. In order to quantify the structure's capacity for flow facilitation, the evolution parameter (*Ev*) is defined as the ratio of the global performance of the evolving configuration (configuration with imperfection) to configuration with least imperfection, under the same global constraints. The evolution parameter varies from zero to one (design with least imperfection) and is defined as follows:
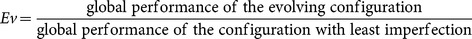
(2)As the evolution parameter approaches one, the configuration evolves to higher flow. The global performance of a flow system is the global flow conductance, which is the reciprocal of the flow resistance. Therefore, the evolution parameter can be expressed in terms of flow resistance as:
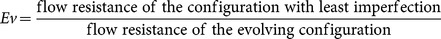
(3)Since the flow resistance is function of flow behavior, the evolution parameter is obtained for different models including fully developed laminar (Newtonian and non-Newtonian fluids) and fully rough turbulent flows.

#### 2.3.1 Newtonian fluids

Although Newtonian fluids such as water are ubiquitous in nature, some non-Newtonian fluids such as blood behave like a Newtonian fluid only at high shear rates (in large blood vessels). For fully developed flows of Newtonian fluids within a symmetrical tree, the global flow resistance 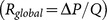, that is the ratio of pressure drop 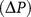 to flow rate 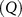, is obtained as (see *S1 Appendix*):
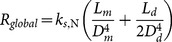
(4)where 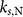 is a function of viscosity, 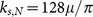. By combining Eqs. (1) and (4), the global flow resistance is written as (see *S1 Appendix*):
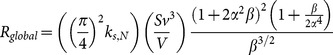
(5)where the shape factors of 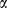 and 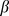 are the diameter ratio 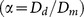 and the length ratio 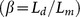, respectively; and 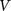 is volume of the tree structure 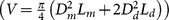. Equation (5) shows that the global flow resistance is a function of fluid properties 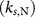, the svelteness, internal size (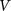) and shape factors (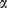 and 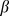). The global flow resistance, Eq. (5), can be minimized with respect to shape factors (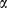 and 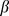). The shape factors that provide the minimal flow resistance are the characteristics of the configuration that facilitates access to the global flow. Minimization of 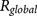 with respect to 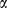 and 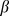, results in the following shape factors (see *S1 Appendix*):
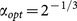
(6)

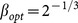
(7)Incidentally, Eq. (6) is the same as Murray's law for symmetrical trees. Equations (6) and (7) can be verified by the results in ref. [26]. Substitution of 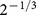 for shape factors in the global flow resistance yields the minimal flow resistance of Newtonian flows. Using Eqs. (3), (5), (6), and (7), the evolution parameter for a tree structure is expressed as:
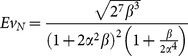
(8)The structure with minimal flow resistance and the evolving structure, which form the evolution parameter, are equal in occupied area and volume, and thus svelteness. Since the evolution parameter is the ratio between the minimal flow resistance and the evolving flow resistance, the evolution parameter is only a function of shape factors for constant properties of fluid 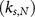.

#### 2.3.2 Non-Newtonian fluids

Most biological fluids such as blood have non-Newtonian behavior (especially in the microvasculature where the shear rate is smaller). For non-Newtonian fluids, viscosity is a non-linear function of shear rate. The power law model is widely used to describe the behavior of a non-Newtonian fluid as:
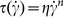
(9)where, 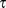 is shear stress; 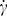 is shear rate; 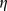 is apparent viscosity and 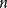 is index of the power law model. An index of the power law describes the shear thinning (pseudo plastic; 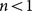) or the shear thickening (dilatant fluids; 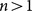) behavior. The flow resistance in a tree structure 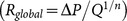 for power law fluids can be written as (see *S2 Appendix*):
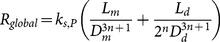
(10)where, 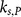 is proportional to the apparent viscosity. For fixed internal and external sizes and constant properties of fluid, the global flow resistance as function of index of the power law model and shape factors is expressed as (see *S2 Appendix*):
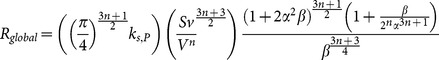
(11)The result of minimizing Eq. (11), as a function of 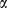 and 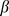, is the same as Newtonian model (Eqs. (6) and (7)), see *S2 Appendix*. Similar to Newtonian model, the svelteness and volume of the evolving structure and the structure with minimal resistance are the same. The evolution parameter is written in terms of shape factors as (see *S2 Appendix*):
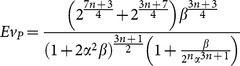
(12)
Equation (12) shows that the evolution parameter is a function of fluid behavior as well as the aspect ratios for non-Newtonian fluids. As expected, the result of Eq. (12) is the same as that of Eq. (8) for 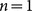.

#### 2.3.3 Turbulent model

Unlike laminar flows where the viscous effects are dominant, turbulent flows are characterized by fluctuant properties due to the non-linear inertia effects. Turbulent flows are common in natural phenomena such as atmospheric circulation, oceanic currents and aortic blood flow. For a tree structure, the global flow resistance for fully rough turbulent flows 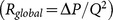 is given by (see *S3 Appendix*):
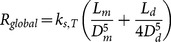
(13)where, 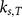 is a function of geometry and fluid properties. The global flow resistance of a tree structure as function of 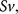 shape factors, volume, and fluid properties is written as (see *S3 Appendix*):
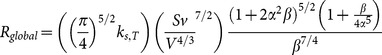
(14)Minimization of 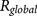 with respect to 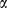 and 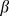, results in:
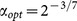
(15)

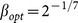
(16)Using Eqs. (3), (14), (15) and (16), the evolution parameter, 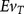, is obtained as (see *S3 Appendix*):
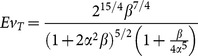
(17)


## Results

### 3.1 Theoretical Results

**Figure 1:**
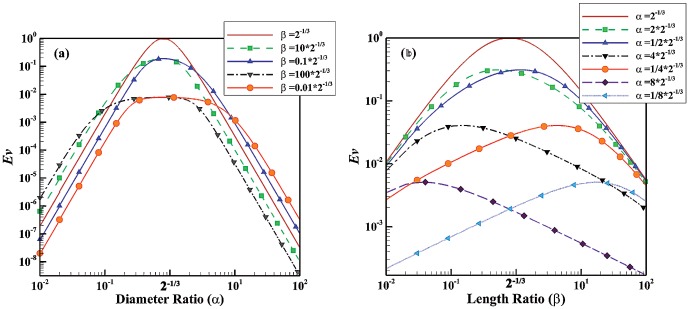
Evolution parameter for Newtonian fluids. (a) *Ev* as function of diameter ratio for different length ratios; (b) *Ev* as function of length ratio for different diameter ratios.

**Figure 2:**
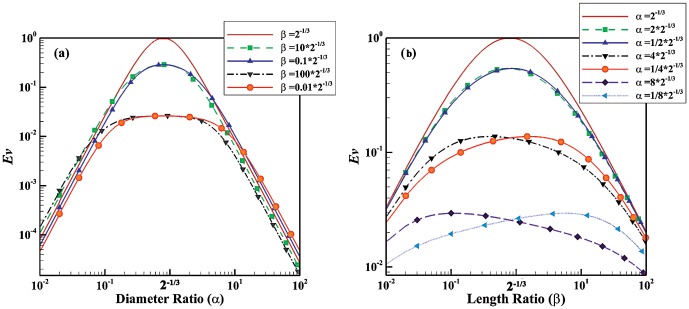
Evolution parameter for non-Newtonian pseudo-plastic fluids with n = 0.5. (a) *Ev* as function of diameter ratio for different length ratios; (b) *Ev* as function of length ratio for different diameter ratios.

**Figure 3:**
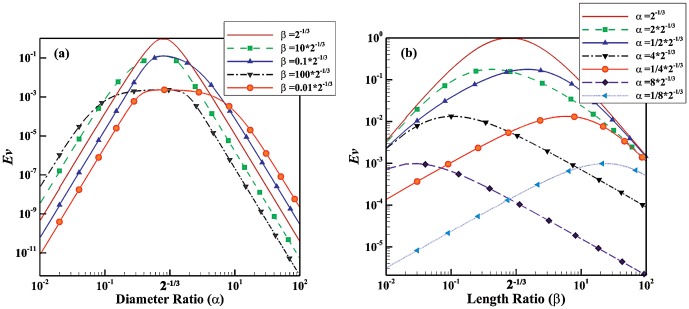
Evolution parameter for non-Newtonian dilatant fluids with n = 1.5. (a) *Ev* as function of diameter ratio for different length ratios;(b) *Ev* as function of length ratio for different diameter ratios.

In order to compare the effect of shape factors on the value of evolution parameter for Newtonian, power law, and turbulent models, *Ev* is plotted as function of 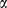 and 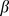. *Ev* is plotted in a wide range of shape factors (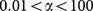 and 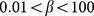). Although the shape factors of vascular structures are nearly unity, we plot *Ev* in a wide range to provide insight into the effect of shape factor on the behavior of the evolution parameter. Fig. 1A shows that for 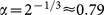 and 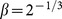, the evolution parameter approaches unity; i.e. the configuration with the least imperfection. Moreover, 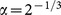 provides the maximum *Ev* at all length ratios. For length ratio other than 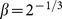, however, the evolution parameter is less sensitive to the diameter ratio. It is also observed that the gradual movement towards 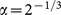 increases the evolution parameter. In addition, very small or large shape factors leads to very small *Ev* (e.g., shape factors equal to 0.01 or 100 results in 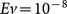 or 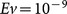, respectively). Fig. 1B demonstrates how the evolution parameter changes when the shape factors approaches 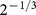. For diameter ratios other than 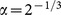, the maximum value of *Ev* oscillates around 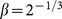. As the diameter ratio approaches 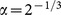, the evolution parameter increases and the maximum *Ev* occurs around 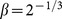. For large values of the diameter ratio (e.g., 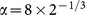), *Ev* reaches a maximum at small values of the length ratio (e.g. 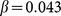). It is noted that for small diameter ratios (e.g., 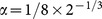), the maximum of *Ev* occurs at relatively large length ratios (e.g. 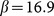). This indicates that a balance between large and small shape factors yields the maximum *Ev*. The evolution parameter is given for non-Newtonian fluids in Figs. 2A,B and 3A,B. Figs. 2A and 2B depict the evolution parameter as function of diameter and length ratios, respectively. The power index of non-Newtonian pseudo-plastic fluid is assumed to be 0.5 (the fluid has a shear-thinning behavior). Figs. 3A and 3B illustrate the evolution parameter as function of diameter and length ratios, respectively for non-Newtonian dilatant fluids with n = 1.5 (the fluid has a shear-thickening behavior). The trend in the evolution parameter for non-Newtonian fluids is similar to that of Newtonian fluids. The value of power index, however, can change the effect of shape factors on the evolution parameter. The shear thinning behavior (e.g. n = 0.5) diminishes sensitivity to the variation of shape factors while the shear thinning effect (e.g. n = 1.5) increases the effect. For example, 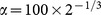 and 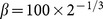 result in 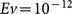 for 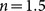, 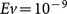 for 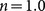, and 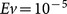 for 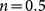. The behavior of *Ev* indicates that the evolution of a flow configuration correlates significantly with the fluid behavior such as non-Newtonian shear thinning or shear thickening behaviors.

**Figure 4:**
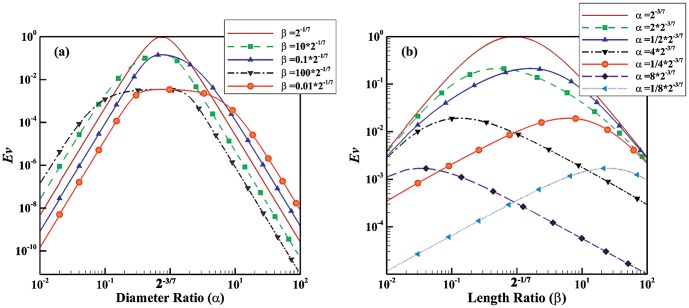
Evolution parameter for a turbulent model. (a) *Ev* as function of diameter ratio for different length ratios; (b) *Ev* as function of length ratio for different diameter ratios.

For a turbulent model, *Ev* is plotted as function of 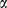 and 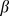 in Figs. 4A and 4B, respectively. The trend in variation of *Ev* is similar to that of laminar flows but maximum *Ev* occurs at 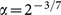 and 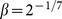 rather. Figs. 4A and 4B show that the flow behavior, such as turbulence, affect the pattern of the evolution parameter alternation. Hence, the flow behavior (laminar or turbulent) as well as fluid behavior (Newtonian and non-Newtonian) change the pattern of *Ev* as function of shape factors.

### 3.2 Anatomical Data

**Figure 5:**
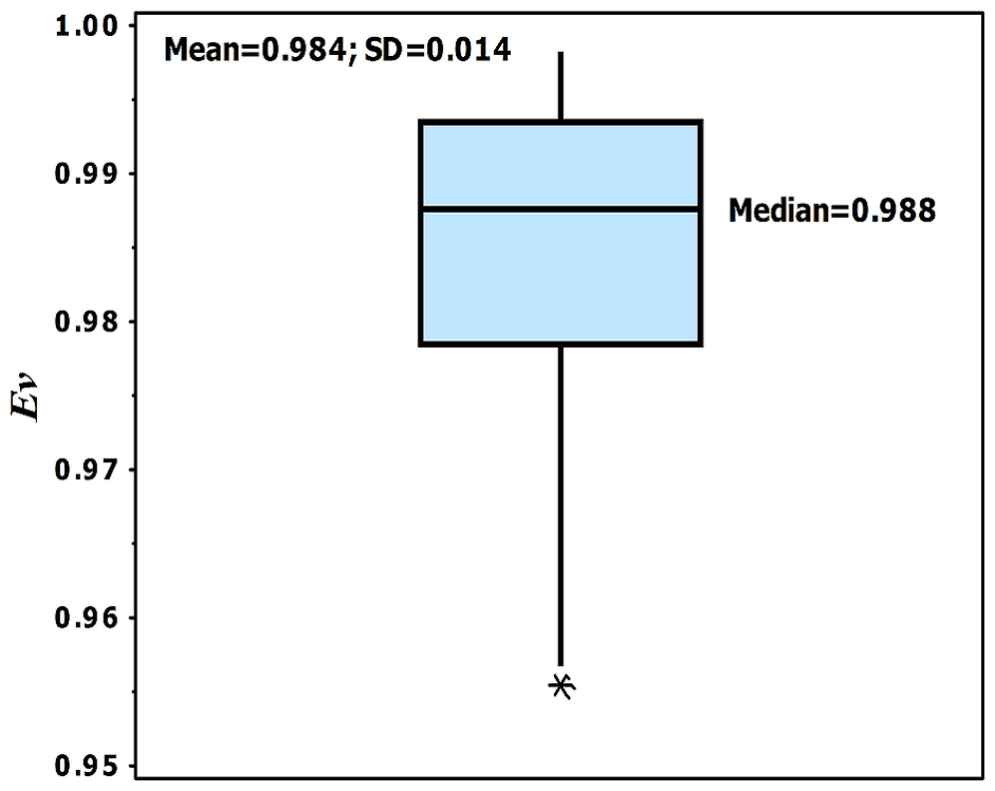
The evolution parameter as function of shape factors. The range of shape factors is obtained from experimental measurement of vascular trees.

The one-third power in Eqs. (6) and (7) generally agrees well with small arteries and arterioles [8], [10]–[12], [28]. Experimental measurement of exponents in 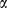 and 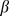, however, show variability for vascular trees of different species and organs [7], [29]–[41]. The anatomical data of various organs and species for entire vascular tree down to the precapillary vessels are used to obtain the shape factors (Table 1). As shown in Table 1, geometrical shape factors have mean ±SD of 0.76±0.04 for the diameter ratio and 0.75±0.04 for the length ratio. Moreover, Fig. 5 shows the evolution parameter for various species and organs. The evolution parameters were calculated by replacing anatomical shape factors in Eq. (8). The median of *Ev* is 0.988 (the structure with the highest flow capacity has *Ev = 1*), and mean value (±SD) equals to 0.984 (±0.014).

**Table 1:** The least-squares of exponents in shape factors 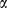 and 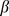, and corresponding *Ev* for different species and organs.

Species 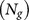	Anatomical data					
	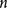	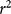	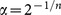	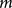	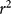	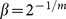
Pig RCA (11)	2.11	0.996	0.72	1.92	0.988	0.7
Pig LAD (11)	2.07	0.993	0.72	1.98	0.990	0.7
Pig LCx (10)	2.04	0.994	0.71	1.8	0.987	0.68
Rat PA (11)	2.22	0.998	0.73	2.14	0.956	0.72
Cat PA (10)	2.37	0.997	0.75	2.33	0.975	0.74
Cat PV (10)	2.3	0.993	0.74	2.18	0.954	0.73
Dog PV (11)	2.5	0.998	0.76	3	0.995	0.79
Human PA (17)	2.65	0.991	0.77	3.16	0.983	0.8
Human PA (15)	2.73	0.994	0.78	3.04	0.978	0.8
Human PA (17)	2.44	0.992	0.75	3.04	0.974	0.8
Human PV (15)	2.65	0.998	0.77	2.92	0.982	0.79
Human PV (15)	2.49	0.994	0.76	2.76	0.986	0.78
Hamster SKMA (4)	2.33	0.992	0.74	2.65	0.87	0.77
Rat MA (4)	3.79	0.990	0.83	2.66	0.924	0.77
Rabbit OV (4)	2.74	0.933	0.78	2.55	0.836	0.76
Human BCA (5)	4.18	0.991	0.85	3.32	0.918	0.81
Human BCV (4)	2.43	0.971	0.75	2.91	0.955	0.79
Hamster RMA (4)	2.05	0.991	0.71	1.71	0.968	0.67
Cat SMA (4)	3.98	0.938	0.84	2.28	0.954	0.74
mean ± s.d.	2.64±0.64	0.76±0.044	2.55±0.49	0.75±0.04		

RCA, right coronary artery; LAD, left anterior descending artery; LCx, left circumflex artery; PA, pulmonary artery; PV, pulmonary vein; SKMA, skin muscle arteries; SMA, sartorius muscle arteries; MA, mesentery arteries; OV, omentum veins; BCA, bulbular conjunctiva arteries; RMA, retractor muscle artery; BCV, bulbular conjunctiva vein; 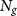, number of total generation in the respective vascular trees [32].

**Figure 6:**
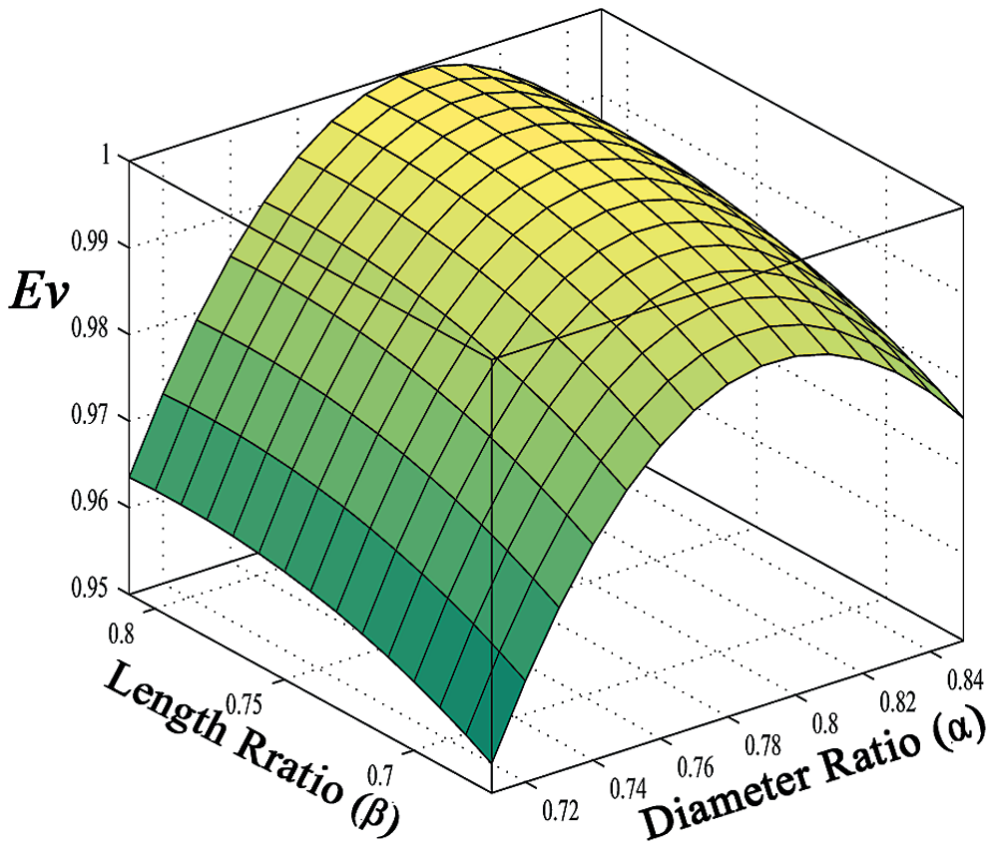
Box and whisker plot of the evolution parameter for various species and organs. The median, mean and SD values are shown in the figure, the box edges represent the 25th and 75th percentiles, the whiskers extend to the most extreme data points not considered outliers. Outliers (star symbols) are larger than P_75_+1.5(P_75_-P_25_) or smaller than P_25_−1.5(P_75_-P_25_), where P_75_ and P_25_ are the 75th and 25th percentiles, respectively.

In order to illustrate the behavior of the evolution parameter within the range of experimental shape factors, a 3-D surface of the evolution parameter is shown in Fig. 6. According to Table 1, experimental shape factors, the diameter and length ratios, varies within the range of 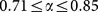 and 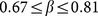, respectively. This figure shows that the evolution parameter is more sensitive to the diameter ratio than the length ratio, and thus the diameter ratio has the major influence on the capacity of the structure for facilitation of flow in the vascular system.

## Discussion

### 4.1 The Significance of The Evolution Parameter

In broad terms, natural selection stipulates that individuals that are more fit have more potential for survival. Since the ultimate goal of vasculature is to nourish tissues, the ability to facilitate flow to transport nutrients within vascular structures may be an evolutionary advantage. A vascular structure that has less flow resistance dissipates less energy, and thus is capable of providing higher flows. Since the vasculature of various species has been subjected to natural selection for flow facilitation, the evolution parameter compares viable alternative designs to the design with highest flow conductance for a given space constraint (e.g., volume). Hence, the evolution parameter assesses the effectiveness of a tree structure to provide higher flow and less energy dissipation.

### 4.2 Evolution Parameter and Murray's Hypothesis

Based on the definition of the evolution parameter, the increase of *Ev* implies a reduction of flow resistance, which provides the capability for higher fluid transport to a limit of space constraints. A higher Ev is consistent with Murray's hypothesis that larger blood vessels demand lager metabolic cost. In summary, a higher *Ev* lowers energy dissipation, and enhances fluid transport for a given constant metabolic cost.

### 4.3 Evolution Parameter and Space Constraints

The evolution parameter is computed in reference to a structure that provides the maximum capacity of flow. Both the evolving and the reference ideal structure have the same constraints (occupied volume and area), and thus the svelteness for both of the structures is equal. Since the occupied volume and area by the structure can change over time, the sveltensss that represents the bulk of the flow structure takes into account the temporal changes in size. Experimental observations also support the relation that mass of tissue scales with the vascular volume that nourishes it. Although the size of tissue may change over time; e.g., during maturation, the fractal principles such as diameter-flow rate, and flow resistance relations govern the design of vasculature. Hence, the evolution parameter can be expressed as a time-independent constant. As shown by Eqs (5), (11) and (14), the flow resistance is a function of flow properties, space constraints, and geometrical shape factors. Since the space constraints are the same as constraints of flow resistance minimization, the geometrical shape factors determine the minimal flow resistance. Hence, the evolution parameter considers the effect of geometrical shape factors on the capacity for flow facilitation, for a given space constraint. In summary, *Ev* parameter indicates the structure's flow capacity while *Sv* parameter represents the structure's size effect.

### 4.4 Effect of Flow Nature on the Evolution Parameter

Blood has a non-Newtonian shear thinning behavior especially in the microvasculature whereas it behaves as a Newtonian fluid in large vessels where shear rate is high. Since biological fluids such as blood have both Newtonian and non-Newtonian behaviors and may behave as laminar (smaller vessels) or turbulent (aorta and heart), the evolution parameter was developed for all the cases. Figs. 1 to 4, indicates that behavior of fluid (Newtonian vs. non-Newtonian), and flow behavior (laminar vs. turbulent regimes) play a major role in the relationship between evolution parameter and geometrical shape factors. For example, the non-Newtonian shear thinning behavior of blood increases the evolution parameter. This implies that non-Newtonian behavior of blood (especially in the microvasculature) leads to higher flow in the vascular system. Since the microvasculature especially arteriolar beds have a significant role in the regulation of blood flow [42]–[45], the higher *Ev* of vascular trees results in higher blood flow in the entire vascular system.

### 4.5 The Evolution Parameter for Various Species and Organs

The evolution parameters in the vascular trees of different species and organs were derived based on measured anatomical data. The mean 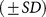 of the shape factors were determined by a least-square fit of morphometric measurement in the vascular trees of different species and organs. Shape factors 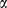 and 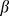 have mean value of 0.76±(0.04) and 0.75±(0.04), respectively. The mean value of shape factors is approximately 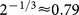, which leads to *Ev* = 1 for both Newtonian and non-Newtonian fluids. As shown in Fig. 6, *Ev* has the mean value of 0.98±(0.01). Hence, the vascular trees in different species and organs facilitate flow within the structure.

### 4.6 Critique and Implications for Future Studies

The major criticism of contstructal theory is that it has not been derived based on first principles [46]. Hence, the existence of global design of macroscopic (finite size) systems that governs evolution is not without controversy. The complication lays in the fact that mechanical forces that determines structural changes act locally rather than globally. To address these issues, Bejan [1], [3] has postulated that the constructal law is a de facto first principle similar to the second law of thermodynamics that stipulates irreversibility. The constructal law postulates a global tendency for flow direction in nature, which implies that constructal design is neither an optimal nor a destiny design.

The proposed evolution parameter measures the design capacity to facilitate flow within the structure from an evolutionary perspective. Since an entire tree structure is comprised of many branches, the evolution parameter was developed for a single branch [47], [48]. As shown by Huo and Kassab [49], the flow resistance of a vessel branch scales with the equivalent resistance of the corresponding distal tree. Kassab and colleagues also have considered the effect of branching structure on hemodynamics considering both symmetric and asymmetric structure [50]–[53]. The major conclusion is that the asymmetry of the network does not significantly affect the mean values but it can significantly affect the spatial heterogeneity of pressure and flow (since by definition a symmetric tree does not have hemodynamic dispersions). Since the current analysis was focused on mean values of pressures and flow, the symmetric assumption is reasonable. Future studies can synthesize the present result of a single branch to the entire tree.

Although the definition of evolution parameter is general, the precise prediction of the flow resistance in the evolution parameter relies on the flow patterns. The Hagen-Poiseuille law (Newtonian and non-Newtonian fluids for laminar flows), and fully rough turbulent flows that formed the basis of analytical formulation neglect the branching and entrance effects. Hence, the flow in 3-D geometry of large bifurcations, which has unsteady nature and complicated patterns such as flow separation, requires computational fluid dynamic (CFD) simulations. Hence, CFD simulations should be utilized in large arteries to provide a basis for formulation of *Ev* in future studies.

## Conclusions

For Newtonian and power law fluids, the same values of the diameter and length ratios of 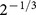 provides *Ev* = 1. However, the diameter ratio of 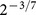 and the length ratio of 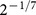 results in *Ev* = 1 for fully rough turbulent flows. Nevertheless, a similar trend was observed for the variation of *Ev* with respect to shape factors. Based on measured anatomical data, *Ev* was found to be approximately one (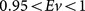) for various organs and species. Since the higher evolution parameter enables the structure to facilitate flow, the results demonstrate that tree structures adapt and evolve in the direction to maximize flow of vascular systems of organs and various species.

## Supporting Information

S1 Appendix **The evolution parameter of Newtonian fluids.**
(DOCX)

S2 Appendix **The evolution parameter for non-Newtonian fluids.**
(DOCX)

S3 Appendix **The evolution parameter for turbulent flows.**
(DOCX)
