# Supplementary material for: Constructal Law of Vascular Trees for Facilitation of Flow
Source: PLoS One. 2014 Dec 31;9(12):e116260. doi: 10.1371/journal.pone.0116260 (PMC4281121; doi:10.1371/journal.pone.0116260)
Supplement: S1 Appendix — The evolution parameter of Newtonian fluids. (DOCX) [file pone.0116260.s001.docx]

**APPENDIX A**

For a Newtonian fluid, the relationship between shear stress and shear rate is linear (). The relationship between flow rate and pressure drop for Newtonian fluids in fully developed laminar regime is expressed as:

| $\Delta p=\left(k_{s,\mathrm{N}}\frac{L}{D^{4}}\right)Q$ | (A.1) |
| --- | --- |

where $k_{s,\mathrm{N}}$ is a constant; $D$ is diameter and $L$ is length of tube. A symmetric tree structure consists of a parent branch with two identical daughter branches at each level (or generation). The total pressure drop is sum of pressure drops in the mother and daughter branches. Since the daughter branches are identical, the pressure drops in mother and daughter branches are the same. Consequently, the total pressure drop $\left(\Delta p_{T}\right)$ is written as:

| $\Delta p_{T}=\Delta p_{m}+\Delta p_{d}$ | (A.2) |
| --- | --- |

where subscripts *m* and *d* denote the mother and daughter branches, respectively. Also, the flow in mother branch divides into two daughter branch. Therefore, the total flow rate $\left(Q_{T}\right)$ in the mother branch is twice the flow rate of daughter branch as follows:

| $Q_{T}=Q_{m}=2Q_{d}$ | (A.3) |
| --- | --- |

Using Eqs. (A.1), (A.2), (A.3), the global flow resistance of a tree structure$\left(R_{global}=\Delta P_{T}/Q_{T}\right)$ is obtained as:

| $R_{global}=k_{s,\mathrm{N}}\left(\frac{L_{m}}{D_{m}^{4}}+\frac{L_{d}}{2D_{d}^{4}}\right)$ | (A.4) |
| --- | --- |

Using the diameter ratio $\left(\alpha=D_{d}/D_{m}\right)$, the length ratio $\left(\beta=L_{d}/L_{m}\right)$, the global flow resistance is rewritten as:

| $R_{global}=k_{s,\mathrm{N}}\frac{L_{m}}{D_{m}^{4}}\left(1+\frac{\beta}{2\alpha^{4}}\right)$ | (A.5) |
| --- | --- |

Similar to the global flow resistance, the svelteness and the volume are expressed in terms of the shape factors as follows:

| $V=\frac{\pi}{4}D_{m}^{2}L_{m}\left(1+2\alpha^{2}\beta\right)$ | (A.6) |
| --- | --- |
| $A=\beta L_{m}^{2}$ | (A.7) |

Using Eqs. (A.5), (A.6), (A.7) and the svelteness$(Sv=A^{1/2}/V^{1/3})$ , the global flow resistance is rewritten as:

| $R_{global}=\left(\frac{k_{s,N}\pi^{2}Sv}{16V}^{3}\right)\left(\frac{\left(1+2\alpha^{2}\times\beta\right)^{2}\left(1+\frac{\beta}{2\alpha^{4}}\right)}{\beta^{3/2}}\right)$ | (A.8) |
| --- | --- |

For constant internal and external size, the volume and svelteness is constant. Therefore, $R_{global}$ is a two-dimensional function of $\alpha$ and $\beta$. According to second-derivative test, at an extremum point $\left(\alpha_{0},\beta_{0}\right)$ the first partial derivatives should be zero, i.e. $\frac{\partial R\left(\alpha_{0},\beta_{0}\right)}{\partial\alpha}=0$ and $\frac{\partial R\left(\alpha_{0},\beta_{0}\right)}{\partial\beta}=0$. Deriving $R_{global}$ with respect to $\alpha$ and $\beta$ results in the following:

| $\frac{\partial R}{\partial\alpha}=0\Rightarrow\alpha=2^{-1/3}$ | (A.9) |
| --- | --- |
| $\frac{\partial R}{\partial\beta}=0\Rightarrow\beta=\frac{1-4\alpha^{6}+\sqrt{16\alpha^{12}+136\alpha^{6}+1}}{12\alpha^{2}}$ | (A.10) |

The second derivative of $\alpha$ and the discriminant $\left(D=\frac{\partial^{2}R}{\partial\alpha^{2}}\frac{\partial^{2}R}{\partial\beta^{2}}-\frac{\partial^{2}R}{\partial\alpha\partial\beta}\right)$ are positive at the minimum point. Using Eqs. (A.8) and (A.9) and (A.10), the shape factors that yield the minimum $R_{global}$ are obtained as:

| $\left(\alpha_{opt}=2^{-1/3},\beta_{opt}=2^{-1/3}\right)$ | (A.11) |
| --- | --- |

Substituting Eqs. (A.11) in Eq. (A.8), yields the minimal global flow resistance. Using Eqs. (3) and (A.8), and the minimal flow resistance, the evolution parameter is obtained as:

| $Ev_{N}=\frac{\sqrt{2^{7}\beta^{3}}}{\left(1+2\alpha^{2}\times\beta\right)^{2}\left(1+\frac{\beta}{2\alpha^{4}}\right)}$ | (A.12) |
| --- | --- |
