# Supplementary material for: Constructal Law of Vascular Trees for Facilitation of Flow
Source: PLoS One. 2014 Dec 31;9(12):e116260. doi: 10.1371/journal.pone.0116260 (PMC4281121; doi:10.1371/journal.pone.0116260)
Supplement: S2 Appendix — The evolution parameter for non-Newtonian fluids. (DOCX) [file pone.0116260.s002.docx]

**APPENDIX B**

For a non-Newtonian fluid, the relationship between shear stress and shear rate is non-linear. The relationship between flow rate and pressure drop of a power law fluid in fully developed laminar regime is obtained as:

| $\Delta p=\left(k_{s,p}\frac{L}{D^{3n+1}}\right)Q^{1/n}$ | (B.1) |
| --- | --- |

where $k_{s,p}$ is a constant and *n* indicates the index of power model. By applying Eqs. (A.2), (A.3), (B.1) similar to Newtonian fluid, the global flow resistance of tree $\left(R_{global}=\Delta P_{T}/Q_{T}^{1/n}\right)$ for power law fluid is obtained as:

| $R_{global}=k_{s,p}\left(\frac{L_{m}}{D_{m}^{3n+1}}+\frac{L_{d}}{2^{n}D_{d}^{3n+1}}\right)$ | (B.2) |
| --- | --- |

Using Eqs. (A.6), (A.7) and (B.2), the shape factors $\left(\alpha,\beta\right)$,the svelteness $(Sv=A^{1/2}/V^{1/3})$, the global flow resistance is expressed as:

| $R_{global}=k_{s,\mathrm{P}}\frac{\pi^{\frac{3n+1}{2}}Sv^{\frac{3n+3}{2}}}{4^{\frac{3n+1}{2}}V^{n}}\left(\frac{\left(1+2\alpha^{2}\times\beta\right)^{\frac{3n+1}{2}}\left(1+\frac{\beta}{2^{n}\alpha^{3n+1}}\right)}{\beta^{\frac{3n+3}{4}}}\right)$ | (B.3) |
| --- | --- |

where $k_{s,\mathrm{P}}$ is a constant. for constant $Sv$ and $V$ the global flow resistance is a function of $\alpha$ and $\beta$. Similar to Newtonian model, the first derivative yields the same results for the diameter and length ratio as:

| $\alpha_{opt}=2^{-1/3}$ | (B.4) |
| --- | --- |
| $\beta_{opt}=2^{-1/3}$ | (B.5) |

Using Eq. (B.3), (B.4) and (B.5), the minimal flow resistance is obtained. Consequently, the evolution parameter for a power law fluid is expressed as:

| $Ev_{p}=\frac{\left(2^{\frac{7n+3}{4}}+2^{\frac{3n+7}{4}}\right)\beta^{\frac{3n+3}{4}}}{\left(1+2\alpha^{2}\times\beta\right)^{\frac{3n+1}{2}}\left(1+\frac{\beta}{2^{n}\alpha^{3n+1}}\right)}$ | (B.6) |
| --- | --- |
