# Supplementary material for: Constructal Law of Vascular Trees for Facilitation of Flow
Source: PLoS One. 2014 Dec 31;9(12):e116260. doi: 10.1371/journal.pone.0116260 (PMC4281121; doi:10.1371/journal.pone.0116260)
Supplement: S3 Appendix — The evolution parameter for turbulent flows. (DOCX) [file pone.0116260.s003.docx]

**APPENDIX C**

For fully developed turbulent flows, flow rate is related to the pressure drop as follows:

| $\Delta p=\left(k_{s,\mathrm{t}}\frac{L}{D^{4}}\right)Q^{2}$ $\Delta p=\left(k_{s,t}\frac{L}{D^{5}}\right)Q^{2}$   | (C.1) |
| --- | --- |

where $k_{s,t}$ is constant. Using Eqs. (A.2), (A.3) and (C.1) for the calculation of global flow resistance results in:

| $R_{global}=k_{s,\mathrm{t}}\left(\frac{L_{m}}{D_{m}^{5}}+\frac{L_{d}}{4D_{d}^{5}}\right)$ | (C.2) |
| --- | --- |

Using the length and diameter ratios, volume and svelteness, Eq. (C2) is expressed as:

| $R_{global}=\left(k_{s,t}\frac{\pi^{5/2}Sv}{4^{5/2}V^{4/3}}^{7/2}\right)\left(\frac{\left(1+2\alpha^{2}\times\beta\right)^{5/2}\left(1+\frac{\beta}{4\alpha^{5}}\right)}{\beta^{7/4}}\right)$ | (C.3) |
| --- | --- |

Solving for the first derivative of $R_{global}$ results in:

| $\frac{\partial R}{\partial\alpha}=0\Rightarrow\alpha=2^{-3/7}$ | (C.4) |
| --- | --- |
| $\frac{\partial R}{\partial\beta}=0\Rightarrow\beta=\frac{3-24\alpha^{7}+\sqrt{576\alpha^{14}+1424\alpha^{7}+9}}{28\alpha^{2}}$ | (C.5) |

Equations. (C4) and (C5) provide the length and diameter ratio at which $R_{global}$ is minimized as follows:

| $\alpha_{opt}=2^{-3/7}$ | (C.6) |
| --- | --- |
| $\beta_{opt}=2^{-1/7}$ | (C.7) |

Similar to Newtonian fluid, applying second derivative test shows the above are the minimum point. Replacing Eq. (C.3) by Eqs. (C6) and (C7) yields the minimal flow resistance. Using minimal flow resistance, Eqs. (C.3) ,and (3), the evolution parameter is expressed as:

| $Ev_{t}=\frac{2^{15/4}\beta^{7/4}}{\left(1+2\alpha^{2}\times\beta\right)^{5/2}\left(1+\frac{\beta}{4\alpha^{5}}\right)}$ | (C.8) |
| --- | --- |
